# Foreign Object Intrusion Detection on Metro Track Using Commodity WiFi Devices with the Fast Phase Calibration Algorithm †

**DOI:** 10.3390/s20123446

**Published:** 2020-06-18

**Authors:** Shuo Li, Jin Xie, Feng Zhou, Weirong Liu, Heng Li

**Affiliations:** 1 School of Electrical and Information Engineering, Changsha University of Science & Technology, Changsha 410114, China; xiejin_96@stu.csust.edu.cn (J.X.); zhoufengcsu@csust.edu.cn (F.Z.); 2 School of Computer Science and Engineering, Central South University, Changsha 410083, China; frat@csu.edu.cn (W.L.); liheng@csu.edu.cn (H.L.)

**Keywords:** foreign objects intrusion detection, CSI, WiFi, indoor localization, phase calibration, angle-of-arrival

## Abstract

With continuous development in the scales of cities, the role of the metro in urban transportation is becoming more and more important. When running at a high speed, the safety of the train in the tunnel is significantly affected by any foreign objects. To address this problem, we propose a foreign object intrusion detection method based on WiFi technology, which uses radio frequency (RF) signals to sense environmental changes and is suitable for lightless tunnel environments. Firstly, based on extensive experiments, the abnormal phase offset between the RF chains of the WiFi network card and its offset law was observed. Based on this observation, a fast phase calibration method is proposed. This method only needs the azimuth information between the transmitter and the receiver to calibrate the the phase offset rapidly through the compensation of the channel state information (CSI) data. The time complexity of the algorithm is lower than the existing algorithm. Secondly, a method combining the MUSIC algorithm and static clutter suppression is proposed. This method utilizes the incoherence of the dynamic reflection signal to improve the efficiency of foreign object detection and localization in the tunnel with a strong multipath effect. Finally, experiments were conducted using Intel 5300 NIC in the indoor environment that was close to the tunnel environment. The performance of the detection probability and localization accuracy of the proposed method is tested.

## 1. Introduction

With the acceleration of urbanization, the metro system is developing rapidly to alleviate the problem of urban traffic congestion [1]. The metro trains operate at high speeds in enclosed tunnels and fixed tracks, making it difficult to avoid obstacles. Therefore, the safe operation requirements of metro trains are extremely high. Foreign objects intrusion on the rail track is one of the crucial issues that seriously affect the safety of trains. Thus, it is necessary to adopt an effective method to achieve the detection and early warning of metro foreign object intrusion. At present, the main extant detection methods for foreign objects in railway tracks include machine vision [2,3,4], passive and active infrared [5,6], and microwave [7,8,9].

However, in the metro tunnel environment, existing methods have some limitations. The machine vision method cannot work well in low light environments such as metro tunnels, and the implementation of machine vision algorithms need complex software and processing requirements. The passive infrared method has the advantages of simple and low power consumption, but its tracking and localization accuracy for intrusive foreign objects is low. Both the active infrared method and the microwave method have high accuracy in detecting foreign objects. However, the detection range of the active infrared method is short. Similar to machine vision, it requires complex software and processing requirements. The microwave method has the problems of complicated hardware and high cost.

In contrast, the WiFi devices, which introduce multiple input multiple output (MIMO) and orthogonal frequency division multiplexing (OFDM) technology under the IEEE 802.11n, have greatly improved signal transmission capability. With the release of open-source 802.11n came measurement and experimentation tools such as Atheros CSI Tool and Linux 802.11n CSI Tool [10,11]. Researchers can extract the detailed physical layer (PHY) wireless communication information from the Atheros WiFi network interface card (NIC), including the channel state information (CSI), the received packet payload, and other additional information (the time stamp, the received signal strength (RSS) of each antenna, the data rate, etc.). Combining the principles of MIMO and OFDM technologies, and using machine learning and signal processing algorithms to process this information, the WiFi device has the ability of environment awareness and target tracking [12,13,14,15,16]. The WiFi networks and devices are ubiquitous, and the price of a network card with a linear array antenna is extremely low. Through simple software modification, the WiFi device can become a foreign object intrusion detection equipment at a low cost. In addition, for foreign object intrusion detection systems for metros, the signal command system for many metro lines is currently based on WiFi technology. For example, in July 2014, the Shenzhen Metro opened a trial run of a WiFi-based mobile Internet system [17]. When implementing a foreign object intrusion detection system based on WiFi technology on such subway lines, the existing WiFi network infrastructure can be used. Only software work is required. Therefore, this solution has cost advantages and high feasibility.

According to the characteristics of foreign objects, there are two methods of RF-signal-based detection: device-free and device-based detection. The device-based detection requires the target to have communication capabilities with RF devices.

Based on the commercial WiFi chip, Spotfi employs the super-resolution angle-of-arrival (AoA). Its estimation achieves high accuracy location. Synchronicity utilizes time synchronization offered by the distributed MIMO network of wireless to locate the target [18,19]. Furthermore, on other devices, Otrack realizes locating and classification by identifying radio frequency identification (RFID) tags on targets [20]. Device-based detection achieves fine-grained localization with high accuracy; however, it relies on the interactions between the signal transmitter and the devices on target objects; in that case, the objects without communication devices are unable to be detected.

Device-free detection can locate unknown targets without communication capabilities. The received signal strength (RSS) was first employed in the device-free detection in an indoor environment; e.g., Nuzzer [21]. Because of the low location accuracy and poor anti-interference of RSS, the CSI from the physical layer replaces the RSS in device-free detection. On the one hand, a high accuracy location algorithm based on CSI was proposed. Pilot and MonoPHY use the CSI from the physical layer to locate the target by building CSI fingerprints [22,23]. CARM builds the CSI-speed model and CSI-activity model based on the good reflectivity of the human body to realize human activity detection [24]. On the other hand, offline training is not essential. LiFS employs pre-processed CSI to build an energy attenuation model to estimate the distance for locating, although it gets a lower location accuracy than the CSI fingerprints methods [25]. Foreign objects intruding in real scenes generally do not have communication capabilities. Therefore, it is reasonable to use device-free detection methods that do not require offline training in the detection of foreign objects in a metro tunnel.

The metro tunnel environment is a complex indoor scene for WiFi-based foreign object detection and localization. To address this problem, our conference paper  [26] proposed a preliminary idea of using WiFi channel state information combined with the MUSIC algorithm to detect foreign objects in subway tunnels and verified the theoretical feasibility with Matlab. However, some challenges must be solved to implement the proposed foreign object intrusion detection method in real scenes using real hardware.

- The MIMO technology introduced by IEEE 802.11n enables the WiFi network card to have the hardware basis for AoA estimation of the target using CSI phase information. However, in a MIMO antenna array, each antenna corresponds to an RF chain. The unsynchronized clocks between the RF chains result in an abnormal random phase difference between different antennas; that is, a phase offset. Therefore, to implement the method proposed in our conference paper on actual hardware, phase calibration must be performed first to eliminate the phase offset. The Phaser [27] is literature that first proposed a method to solve the phase offset of WiFi NICs, but its algorithm is complicated, and the calibration time is long. In Section 3.2, we give the analysis and comparison of the time complexity of our proposed algorithm and the algorithm. The literature  [28] proposed a simple phase calibration method. However, this paper only sets the scene containing a single device-based target to verify the effectiveness of the proposed method. At the same time, the phase offset value they observed is fixed at π, which may be true under certain conditions. However, our experiments found that this phase offset value is random.
- The detection and tracking of foreign objects are achieved by acquiring and analyzing the reflected signals caused by foreign objects. However, in real scenes, besides the line of sight (LOS) signal path between the receiver and the transmitter, and the reflection path caused by intruding foreign objects, there are also many reflection paths caused by walls and non-target objects. The existence of these reflection paths seriously affects the detection and tracking of intruding foreign objects. Therefore, ensuring the accuracy of foreign object detection and tracking in a real scene with multiple signal paths is one of the core problems that needs to be solved.

To address these challenges, we propose a static clutter suppression MUSIC algorithm based on fast phase calibration to achieve foreign object detection in tunnel environments. The fast phase calibration algorithm efficiently realizes the phase offset correction, and the static clutter suppression reduces the influence of the non-target reflected signal path on the foreign object detection in the tunnel scene. Compared with the existing research and the author’s published conference paper [26], this paper’s main contributions are summarized as follows:

1. Fast phase calibration algorithm. A fast phase calibration algorithm is proposed based on the law of phase offset observed from the results of extensive experiments. The algorithm calculates the CSI phase offset matrix based on the AoA information of the direct path between the transmitter and the receiver to implement automatic phase calibration. Compared with other phase calibration algorithms, this algorithm has a lower time complexity, which is O(M)+O(N); M is the number of antennas, and N is the number of subcarriers. The theoretical basis for phase calibration based on CSI data is given in Section 3.2.
2. MUSIC algorithm based on static clutter suppression. Based on the in-depth analysis of the change of radio frequency signals in the process of foreign objects intrusion, using the non-coherence between the static path signal and the dynamic reflection signal caused by the foreign objects intrusion, a MUSIC algorithm without spatial smoothing is proposed. Without spatial smoothing, static path signals that are coherent are superimposed into one signal, thereby significantly reducing the total number of signals (paths) and improving the efficiency of foreign object detection and tracking. Simultaneously, combined with static clutter suppression technology, the algorithm’s adaptability to the environment is enhanced.
3. The intrusion detection system is implemented with the commercial intel 5300 WiFi NIC, and only a pair of transceivers are required. That is a system that integrates sensing and communication. When a foreign object is detected, the intrusion information can be sent to the control center through the communication link.

The remainder of this paper is organized as follows: Section 2 presents the system architecture of the foreign object intrusion detection in metro. The CSI data preprocessing methods, including fast phase calibration, are presented in Section 3. Section 4 presents the location method by MUSIC algorithm based on static clutter suppression. In Section 5, the performance of the proposed method is analyzed and verified by presenting numerous experiment results, and followed by the conclusions and future works in Section 6.

## 2. System Architecture

**Figure 1:**
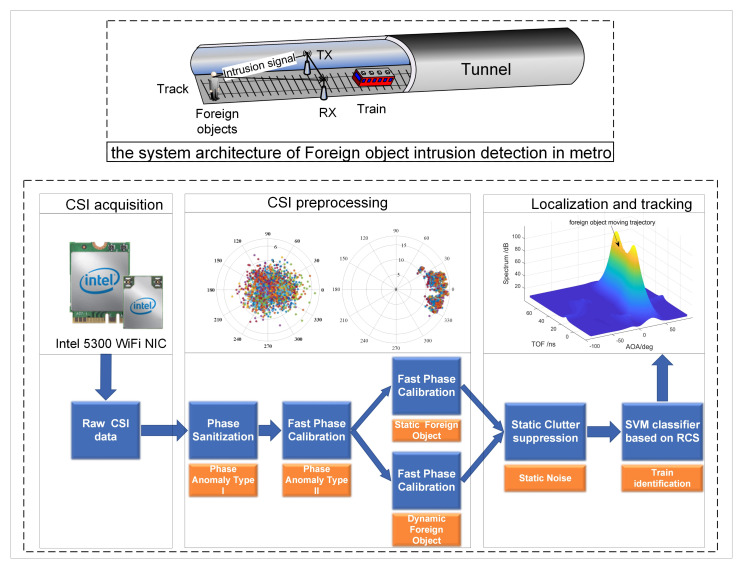
System architecture of foreign object intrusion detection in metro.

The architecture and signal processing flow of the WiFi-based subway foreign body intrusion detection system is shown in Figure 1. The system’s basic principle is to find and locate foreign objects by analyzing the effects of foreign objects on the propagation of radio frequency signals. The system is divided into three main modules: CSI acquisition, CSI preprocessing, foreign object positioning, and tracking. First, the original CSI data with phase abnormality is obtained through the CSI acquisition module. Second comes inputting the original CSI data to the CSI preprocessing module to correct the phase abnormality. Finally, a positioning algorithm is used to process the corrected CSI data in real-time to estimate the position of foreign objects, and a clutter suppression algorithm is used to improve the robustness of the algorithm. The detailed description of each module is as follows:

1. CSI acquisition. CSI could provide detailed channel frequency response information on multiple channels in the physical layer. In our system, Intel 5300 NIC and CSI Tool firmware [10,11] are used to obtain CSI. However, because the clock is not synchronized, there is a phase abnormality in the raw CSI data collected directly, which cannot be used by the positioning algorithm.
2. The CSI preprocessing module implements the correction of the phase anomalies in the raw CSI data. There are two types of phase anomalies.
  **Phase anomaly type I.** The first type of phase anomaly is caused by the unsynchronized clock between the receiver and the transmitter. The phase sanitization algorithm in SpotFi [18] is used to correct it.
  **Phase anomaly type II.** The second type of phase anomaly is caused by the unsynchronized clocks between the receiver’s RF chains and is called an abnormal phase offset. It can be corrected by the fast phase calibration algorithm proposed in this paper. The algorithm is described in detail in Section 3.
3. Localization and tracking of foreign objects. In this module, based on the MIMO and OFDM technologies of the Intel 5300 NIC, the MUSIC algorithm is used to process the calibrated CSI data to realize the estimation of the AoA and distances of foreign objects, and to locate and track the foreign objects in real-time. A spatial smoothing strategy for scene matching is proposed to improve the utilization of sensors (antennas and subcarriers) through an in-depth analysis of the relationship between the motion state of foreign objects and signal coherence efficiency of foreign object detection. Besides, by introducing static clutter suppression technology, the algorithm’s anti-interference and environmental adaptability are improved. Finally, the radar cross section (RCS) data set of foreign objects and trains is constructed based on simulation, and the classification of foreign objects and trains is realized by using the SVM algorithm. The detailed explanation is in Section 4.

## 3. Cause and Calibration Method of Phase Abnormality

As mentioned in Section 2, the original CSI data must be preprocessed because of the abnormal phase. The phase anomaly type I is caused by the unsynchronized clocks between the transceivers. The method described in Appendix A can be used to correct the anomalies. This section analyzes the causes of phase anomaly type II and proposes a fast phase calibration algorithm to calibrate this type of phase anomaly.

### 3.1. Phase Anomaly Induced by Clock Non-Synchronization between RF Chains on a WiFi NIC

The three antennas at intel 5300 NIC are uniformly arranged at a spacing of *d*. In theory, when the detection target is stationary, the phase difference between two RF chains is −2π×d×sin(θ)×f/c, where θ is the signal incident angle and *f* is the subcarrier center frequency.

In the real experiment, in an outdoor open space, we keep the angle between the receiver and the transmitter at 5 degrees and the distance of 6 m. The transmitter is equipped with a single antenna, the receiver is equipped with three antennas, and the antenna 2 is in the middle position. We perform 200 experiments, and each experiment sends 100 packets from the transmitter to the receiver, and the time interval between the packets is 10 ms.

**Figure 2:**
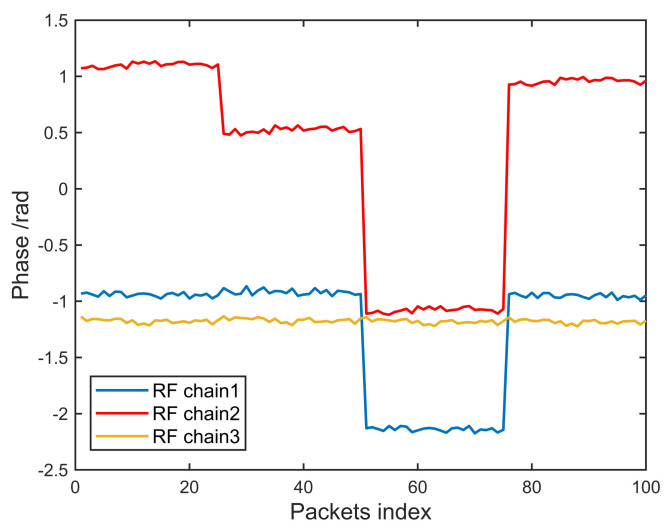
Phase offsets of the 15th subcarrier.

After phase sanitization, the phase of the 15th subcarrier of each antenna is plotted with the packet index as the horizontal axis coordinate. The results of 200 experiments are almost the same, as shown in Figure 2.

Based on this extensive CSI data collection and analysis experiment, we discovered the law of phase offset between receiver RF chains. First, because the angle between the receiver and the transmitter is fixed at 5 degrees, and the experiment is conducted in an open outdoor environment, the phase difference between the RF chains should be small and kept constant. As shown in Figure 2, the experimental results show that the phase difference between the RF chains is not constant, but changes randomly. We consulted relevant literature to explain this phenomenon. As shown in [29], a WiFi network card with MIMO capability is composed of a master chip and several slave chips. Each slave chip is responsible for processing signals on an RF chain, and different PLLs drive the slave chips. Because the clock signal output by the PLL cannot achieve nanosecond-level synchronization, different slave chips generate additional abnormal phase offset when processing the same received signal.

This phase offset is disadvantageous to the AoA estimation of foreign objects based on the MUSIC algorithm. However, a useful feature is that the phase offset only occurs on one RF chain simultaneously, and there has never been a case where more than two RF chains have a phase offset at the same time. For example, from the first packet to the 46th packet, there is phase offset on chain 2, and the phase of chain 1 and chain 3 are not offset. From the 47th to 78th packets, the phase of chain 1 is offset, and the phases of chain 2 and chain 3 are normal. This characteristic of phase offset makes it possible to achieve phase calibration rapidly. In the next section, we give the theoretical basis for phase calibration and then provide the specific implementation of the fast phase calibration algorithm.

### 3.2. Fast Phase Calibration Algorithm

To eliminate the phase offset between RF chains, this section proposes the fast phase calibration algorithm. This section first describes the theoretical basis of the algorithm and then gives the specific implementation of the algorithm.

**Figure 3:**
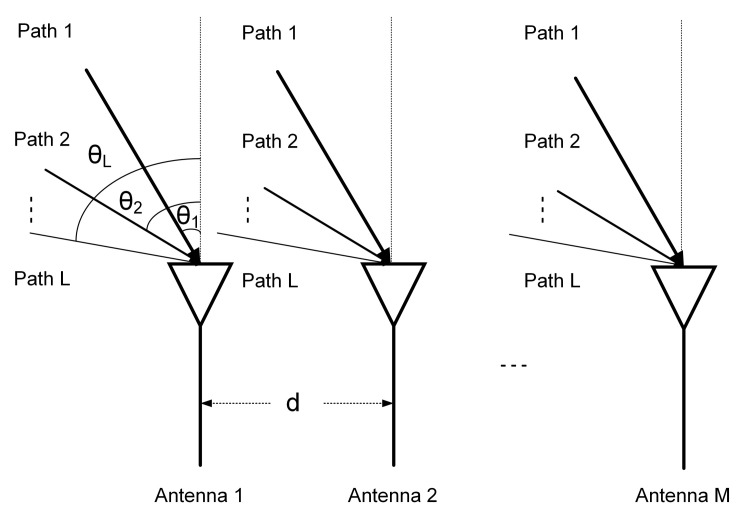
Uniform linear array of M receiving antennas.

It is assumed that in a tunnel environment, WiFi signals propagate on L paths due to reflections of foreign objects and other non-target objects, and the LOS path between the receiver and the transmitter. On the *k*th propagation path, the signal is incident on the uniform line array composed of *M* antennas in Figure 3 at an angle $\theta_{k}$, and the interval between the antennas is *d*. In the real experimental environment, there are three receiving antennas on the intel 5300 WiFi NIC; i.e., M=3. Suppose S(t) represents the transmitted signal on all *L* propagation paths:

(1) $$S\left(t\right)={\left[S_{1}\left(t\right)\;S_{2}\left(t\right)\;\cdots \;S_{L}\left(t\right)\right]}^{T}$$

A(θ) represents the ideal steering matrix for transmitting signals on all *L* propagation paths:

(2) $$A\left(\theta \right)=\left[\begin{array}{cccc} 1 & 1 & \cdots & 1\\ e^{-j\phi_{1}} & e^{-j\phi_{2}} & \cdots & e^{-j\phi_{L}}\\ e^{-j\left(M-1\right)\phi_{1}} & e^{-j\left(M-1\right)\phi_{2}} & \cdots & e^{-j\left(M-1\right)\phi_{L}} \end{array}\right]$$

where $\phi_{k}=-2\pi d\sin\left(\theta_{k}\right)\lambda$ is the phase offset of the transmitted signal on the first antenna of the uniform linear array, λ is the signal wavelength; the *k*th column represents the phase function of the three antennas on the *k*th propagation path, k∈1,2…L.

In the OFDM technology, data are propagated over *N* subcarriers. On the *k*th propagation path, transmitting signal is:

(3) $$S_{k}\left(t\right)=\left[s_{1}\left(t\right)\;s_{2}\left(t\right)\;\cdots \;s_{N}\left(t\right)\right]$$

The subcarriers are introduced with a phase difference $\psi_{k}=2\pi \times f_{\delta }\times \tau_{k}$ after the time of flight (ToF) $\tau_{k}$ from the subcarriers, where $f_{\delta }$ is the frequency interval of adjacent subcarriers. Let $\Phi_{\theta k}=e^{-j\phi_{k}}$ and $\psi_{\tau k}=e^{-j\psi_{k}}$; then the steering matrix of the *k*th path can be expressed as:

(4) $$A\left(\theta_{k},\tau_{k}\right)=\left[\begin{array}{cccc} 1 & \psi_{\tau k} & \cdots & \psi_{\tau k}^{N-1}\\ \Phi_{\theta k} & \Phi_{\theta k}\psi_{\tau k} & \cdots & \Phi_{\theta k}\psi_{\pi }^{N-1}\\ \vdots & \vdots & \ddots & \vdots \\ \Phi_{\theta k}^{M-1} & \Phi_{\theta k}^{M-1}\psi_{\tau k} & \cdots & \Phi_{\theta k}^{M-1}\psi_{\tau k}^{N-1} \end{array}\right]$$

The multipath signal and noise n(t) on all RF chains are superimposed to form the ideal CSI matrix:

(5) $$\mathrm{CSI}=\left[A\left(\theta_{1},\tau_{1}\right)\;A\left(\theta_{2},\tau_{2}\right)\cdots \;A\left(\theta_{L},\tau_{L}\right)\right]\left[\begin{array}{c} S_{1}\left(t\right)\\ S_{2}\left(t\right)\\ \vdots \\ S_{L}\left(t\right) \end{array}\right]+n\left(t\right)$$

However, in actual hardware, because the clocks of the NICs’ RF chain are not synchronized, the phase offset $e^{{-j\delta }_{m}}$ is induced on the RF chain. Thus, a phase offset matrix on the antenna array is O(δ):

(6) $$O\left(\delta \right)=\left[\begin{array}{cccc} e^{-j\delta_{1}} & 0 & \cdots & 0\\ 0 & e^{-j\delta_{2}} & \cdots & \vdots \\ \vdots & \vdots & \ddots & 0\\ 0 & 0 & \cdots & e^{-j\delta_{M}} \end{array}\right]$$

Then the actual ${\mathrm{CSI}}_{re}$ matrix should be the result of multiplication with the phase offset matrix, ${\mathrm{CSI}}_{re}=O\left(\delta \right)\times \mathrm{CSI}$.

If all RF chains have a phase offset, that is, $e^{{-j\delta }_{m}}\neq 0$ for $\forall m\in \left\{1,2,\ldots ,M\right\}$, the matrix data are difficult to obtain. However, according to the experiments in Section 3.1, it is found that only one RF chain has a phase offset at any time so that it is possible to obtain the matrix O(δ). The goal of the fast phase calibration algorithm is to calculate the phase offset according to the AoA information of the LOS path between the receiver and the transmitter, find the phase offset chain and correct it, and finally obtain the CSI matrix without phase offset.

**Figure 4:**
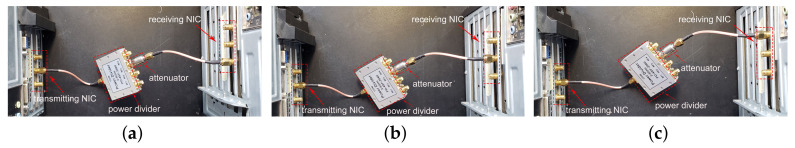
The connection of the antenna port of the transmitting NICand the receiving NIC. (**a**) transmitting port2 connects receiving port1. (**b**) transmitting port2 connects receiving port2. (**c**) transmitting port2 connects receiving port3

According to Figure 4 and Section 3.2, when the distance *d* between the antennas is constant, the phase difference between the RF chains is a function of the AoA of the propagation path. Given the AoA of the LOS path, the theoretical value of the phase difference between RF chains can be calculated. By comparing the theoretical and actual values, the RF chain that induced the phase offset can be found. The key issue is to find the mapping relationship between the three logical RF chains and the three antenna ports.

The CSI Tool generates a CSI-entry for each packet. The CSI-entry contains a 1×3×30 CSI matrix and an attribute named perm. The attribute perm indicates how the NIC permuted the signals from the three receive antennas into the three RF chains that process the measurements. For example, the value of perm$\left[3\;2\;1\right]$ implies that Antenna C was sent to RF Chain A, Antenna B to Chain B, and Antenna A to Chain C. This operation is performed by an antenna selection module in the NIC and generally corresponds to ordering the antennas in decreasing order of RSS.

According to this characteristic, we use an attenuator, a power divider, and two coaxial RF lines to find the mapping relationship between the antenna and the RF chain. As shown in Figure 4, one end of the coaxial cable is connected to one antenna port of the transmitting NIC, and the other end of the cable is sequentially connected to three antenna ports of the receiving NIC, because the RSS on the antenna port connected with the coaxial cable is much higher than the other two ports. By analyzing the changes in the value of the perm attribute, the mapping relationship between the RF chains and the antenna ports can be obtained, as shown in Table 1. We summarize the complete fast phase calibration algorithm in Algorithm 1. By analyzing the fast phase calibration algorithm, we can calculate that the time complexity of the core part of the algorithm is O(M)+O(N), where *M* is the number of antennas (M=3), and *N* is the number of subcarriers (N=3). The Phaser’s [27] time complexity is O(L*S), *L* is the number of antennas, and *S* is the number of calibration candidate populations. To obtain the same AoA estimation accuracy as the algorithm proposed in this paper, *S* must be greater than or equal to 32.
**Algorithm 1** The fast phase calibration algorithm.**Input:** the AoA of the LOS path θ, raw CSI matrix obtained by the CSI Tool [10], antenna spacing *d*, subcarrier center frequency *f***Output:**${\mathrm{CSI}}_{re}$ matrix 1:Read the mapping relationship Table 1.2:Calculate the theoretical phase difference $\varphi_{t}=-2\pi \times d\times \sin\left(\theta \right)\times f/c$3:unwrap: ψ = unwrap(angle(CSI))4:**for** subcarriers n∈1,2…N(N=30) **do**5: Calculate the actual phase difference $\varphi_{12},\varphi_{13},\varphi_{23}$ between the RF chains6: Compare $\varphi_{t}$ and $\varphi_{12},\varphi_{13},\varphi_{23}$ to find the phase offset chain *m*7: Calculate phase offset $e^{{-j\delta }_{m,n}}$8:**end for**9:Calculate $e^{{-j\delta }_{m}}$ using the least squares algorithm10:Calculate the phase offset matrix O(δ) in Equation  (6)11:Return:${\mathrm{CSI}}_{re}$ matrix without phase offset

**Figure 5:**
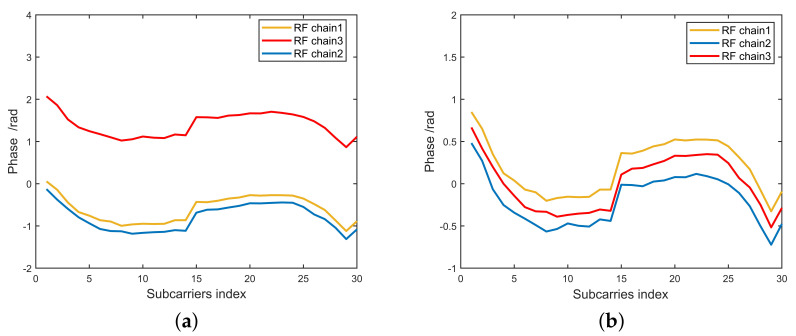
Unwrapped CSI phase. (**a**) Unwrapped CSI phase with phase sanitization, without phase calibration. (**b**) Unwrapped CSI phase with phase sanitization and phase calibration.

To test the fast phase calibration algoritm, the receiver and transmitter are placed outdoors at an angle of 5 degrees. The actual and calibrated CSI phase responses for the same packets obtained from CSI Tool collected from our experiments are presented in Figure 5. Figure 5a is experimental result without phase calibration. As shown in Figure 5a, the RF chain3 phase offsets, resulting in incorrect path number and angle estimates. Figure 5b is the experimental result after phase calibration. As can be seen from Figure 5b, the phase offset of RF chain3 is corrected, and the angle of LOS path between receiver and transmitter is estimated to be 5 degrees, which is the same as the actual angle in the experiment.

## 4. Localizing the Foreign Object

The CSI data without phase anomaly problem are sensitive to the foreign objects’ location; therefore, we must use a reasonable algorithm to accurately locate the target by the CSI data.

It is a typical device-free localization problem to detect foreign objects in metro tunnels. In this paper, WiFi technology is used to achieve the target location, which relies on the radio frequency (RF) techniques and the existence or movement of foreign objects’ assumption that will, in turn, disrupt the original RF models.

**Figure 6:**
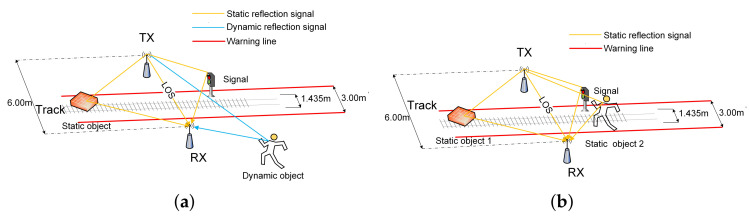
Foreign objects intrusion model. (**a**) Scene of foreign object movement. (**b**) Scene of foreign object static.

Firstly, as shown in Figure 6a, in the tunnel environment, the state of foreign object intrusion is from motion to static. In this process, there are two types of signals according to the differences in signal propagation paths. One type is the static path signal. This type of signal includes the signal propagating on the LOS path (direct path) between the receiver and the transmitter, and the signal propagating on the reflection path caused by static objects (for example, the signal near the track, the wall, etc.). The signals are coherent with each other. The MUSIC algorithm perceives the distinct coherent signals as one superimposed signal, so it is impossible to distinguish these signals using the traditional MUSIC algorithm.

Another type of signal is a dynamic path signal that propagates on a dynamic path caused by moving foreign objects. The dynamic path signal and the static path signal are mutually incoherent signals. The traditional MUSIC algorithm can distinguish incoherent signals, so that it is easy to detect the dynamic path caused by foreign objects and locate foreign objects. Using this coherence, the number of signals to be processed at the receiving end is greatly reduced, thereby greatly improving the sensor’s utilization efficiency. For example, for a receiver with three receiving antennas and 30 subcarriers, theoretically, it is possible to detect 3×30−1 dynamic path signal signals. However, in practical applications, the number of paths that can be detected is much smaller than the theoretical value due to the influences of calibration, noise, and especially the distribution density of foreign objects.

Secondly, another scenario is shown in Figure 6b, when the foreign object completes the intrusion process and changes from the motion state to the stationary state. In the scene, all signals are static path signals and are coherent signals with each other. The spatial smoothing algorithm needs to be used to pre-process the CSI matrix, and then the MUSIC algorithm can be used to estimate the information of the reflection path caused by Static foreign objects. The method for constructing a smoothed CSI matrix from the raw CSI matrix is shown in Appendix C. Constructing a smoothed CSI matrix can achieve signal decoherence, but it will reduce the utilization efficiency of the sensor (antenna, subcarrier).

### 4.1. Propagation Paths Number and Super-Resolution AoA Estimation

In the tunnel environment, the WiFi signal propagates through a direct path and reflection path. The intrusion of foreign objects will increase the number of reflection paths. If the foreign object is in a stationary state, the signals of these reflection paths are coherent, and the coherence signals are decohered using a smoothing technique. The MUSIC algorithm and the optimal information theory criterion are used to estimate the number of propagation paths accurately. By comparing the number of propagation paths before and after foreign object intrusion in real-time, the existence of foreign objects can be judged. Furthermore, the spectrum peak search is performed by calculating the spatial spectrum function to estimate the AoA of the foreign object.

First, construct the covariance matrix R, as shown in Equation (7), where X is the ${\mathrm{CSI}}_{re}$ matrix obtained by performing phase calibration on the raw CSI. The calculation method of the ${\mathrm{CSI}}_{re}$ matrix is shown in Equations (5) and (6). The matrix X needs to be spatially smoothed if the received signals are coherent, according to the method shown in Appendix C. Because the signal and noise are independent of each other, the covariance matrix R can be decomposed into two parts—signal and noise, where $R_{S}$ is the signal covariance matrix and $AR_{s}A^{H}$ is the signal part, where A is the steering matrix in Equation (4).

(7) $$R=E\left[{XX}^{H}\right]=AR_{S}A^{H}+\sigma^{2}I$$

The eigen-decomposition of R is as Equation (8), where $U_{S}$ is a signal subspace constructed by the eigenvectors corresponding to the largest *a* eigenvalues, and $U_{N}$ is a noise subspace constructed by the eigenvectors corresponding to the smallest A−a eigenvalue.

(8) $$R=U_{S}\Sigma_{S}U_{S}^{H}+U_{N}\Sigma_{N}U_{N}^{H}$$

*a* is the number of the largest eigenvalues; that is, the number of paths of WiFi signal propagation. The value of *a* is estimated using the information theory criterion. The information theory criterion proposed by WaxM and KailatT in the literature [30,31] includes effective detection (EDC), Akaike information theory criteria (AIC), and minimum description length criteria (MDL).

The calculation method of each criterion is shown in Appendix B. Accurate path number estimation is an essential basis for realizing foreign object detection. Therefore, it is necessary to determine which criterion has better performance in the current experimental environment. Based on the experimental comparison, it is proven that the hannan-quinn (HQ) criterion has the highest accuracy of path number estimation. The specific experimental results are shown in Section 5.1.1.

After the path number estimation is completed, the angle estimation is performed. Ideally, the $U_{S}$ and the $U_{N}$ are completely orthogonal, and it means the steering matrix of the signal subspace is orthogonal to the noise subspace:

(9) $$A^{H}\left(\theta \right)U_{N}=0$$

In practice, the number of receiving data packets is limited. So the maximum likelihood estimation of covariance matrix R is expressed as:

(10) $$\hat{R}=\frac{1}{Z}\sum_{i}^{Z}{XX}^{H}$$

where Z is receiving data packets. In addition, the $U_{S}$ and $U_{N}$ are not completely orthogonal because of the noise. As a result, the AOA is estimated by the minimum optimal search:

(11) $$\theta_{MUSIC}=\underset{\theta }{\arg\min}A^{H}\left(\theta \right){\hat{U}}_{N}{\hat{U}}_{N}^{H}A\left(\theta \right)$$

The spatial spectrum function of the MUSIC algorithm is expressed as:

(12) $$P_{MUSIC}=\frac{1}{A^{H}\left(\theta \right){\hat{U}}_{N}{\hat{U}}_{N}^{H}A\left(\theta \right)}$$

Once there are coherent signals, the rank of signal subspace $U_{S}$ is not full. It results in the dimension of $U_{S}$ being smaller than $U_{N}$, which cannot be orthogonal so that AOA cannot be correctly estimated. Thus, it is necessary for $U_{S}$ of coherent signals to obtain a full rank. We use the way of spatial smoothing to decoherent R from literature  [18]. Spatial smoothing is achieved by reconstructing the CSI matrix. The specific method is shown in Appendix C. Spatial smoothing can achieve decoherence, but it reduces the efficiency of the sensor (antenna).

### 4.2. Target Distance and Location Estimation

Since super-resolution AoA estimation offers the reliable AOAs of foreign objects, what we need to locate the foreign objects are the distances from foreign objects to the receiver. In this way, we can overcome the disadvantage of location methods based on fingerprints that off-line training is required. Moreover, the fingerprints method has a good performance just for one target, while there could be more than one target intruding into the metro system [32,33].

In general, the RSS from the physical layer is used to estimate the distance, resulting in a low estimation accuracy because of its poor anti-interference of multipath environment. Therefore, we estimate the distance by using the CSI energy attenuation from literature [25], which suggests that CSI is sensitive enough to the target, and by modeling the CSI energy attenuation, the distance can be estimated. Thus, locating foreign objects can be expressed as the following steps:

1. Distance estimation. The use of the CSI amplitude as the signal energy attenuation to construct the distance-energy attenuation model. There are three kinds of energy attenuation from transmitter *i* to receiver *j* of signals in wireless communications: propagation attenuation $L_{ij}$, diffraction attenuation $D_{ij}$, and target *t* absorption attenuation $A_{t}$. All the $L_{ij}$, $D_{ij}$, and $A_{t}$ are the functions of the distance $d_{i,t}$ from transmitter *i* to target *t* and the distance $d_{j,t}$ from target *t* to receiver *j*. Thus, the amplitude attenuation of CSI denoted by $R_{ij}$ can be expressed by:
  (13) $$R_{i,j}\left(d_{i,t},d_{j,t}\right)=\left\{\begin{array}{cc} L_{i,j}\left(d_{i,t},d_{j,t}\right)+D_{i,j}\left(d_{i,t},d_{j,t}\right)+A_{t}\left(d_{i,t},d_{j,t}\right),\;\;\; & NLOS\\ D_{i,j}\left(d_{i,t},d_{j,t}\right)+A_{t}\left(d_{i,t},d_{j,t}\right),\;\;\; & LOS \end{array}\right.$$
  The non line of sight (NLOS) in Equation (13) denotes that the foreign object appears in the NLOS path, while LOS denotes that the foreign object appears in the LOS path. Thus, the amplitude attenuation of CSI $R_{ij}$ is a function of $d_{i,t}$ and $d_{j,t}$, $R_{ij}=f\left(d_{i,t},d_{j,t}\right)$. Then we get the distance $d_{j,t}$ to locate the foreign objects.
2. Calculate foreign object coordinates. We use the signal receiving as the origin to establish the two-dimensional plane coordinate system where the angle of the foreign object repoint relative to the origin is $\theta_{t}$, and the distance is $d_{j,t}$. Thus, the coordinates of the foreign object P(x,y) will be uniquely determined, where the x-coordinate $x=d_{j,t}\cdot \cos\theta_{t}$ and the y-coordinate $y=d_{j,t}\cdot \sin\theta_{t}$.

### 4.3. Static Clutter Suppression

In addition to the localization of static foreign objects, it is also necessary to locate the coordinates of foreign objects in real-time and continuously in the dynamic process of foreign object intrusion. The coordinate calculation of foreign objects in the intrusion process can be used to judge the relationship between the intrusion route and the warning area. In the tunnel environment, in addition to the signal reflection path induced by the intrusion of foreign objects, there are also direct paths between the receiver and the transmitter and reflection paths induced by other non-target objects. The existence of these signal paths obviously has a negative impact on foreign object detection. However, these paths are all static paths, and the signals propagating on the static paths are coherent. Therefore, these signals can be reduced by static clutter suppression algorithms.

The MUSIC algorithm generates a pseudo-spectrum for each packet. The pseudo-spectrum can be regarded as a matrix containing the distance and angle information of the target. The static clutter suppression algorithm is implemented by subtracting from the samples the mean value of the matrix. Therefore, static clutter suppression is a cross packet algorithm. As shown in Equation (14), $P_{MUSIC,t}$ is the pseudo-spectrum at time t, and ${\hat{P}}_{MUSIC,t}$ is the new pseudo-spectrum after static clutter suppression.

(14) $${\hat{P}}_{MUSIC,t}=\left\{\begin{array}{cc} P_{MUSIC,t}-\left(\left\{\sum_{t=1}^{T}P_{MUSIC,t}\right\}/T\right),\;\;\; & {\hat{P}}_{MUSIC,t}\geq 0\\ 0,\;\;\; & {\hat{P}}_{MUSIC,t}<0 \end{array}\right.$$

**Figure 7:**
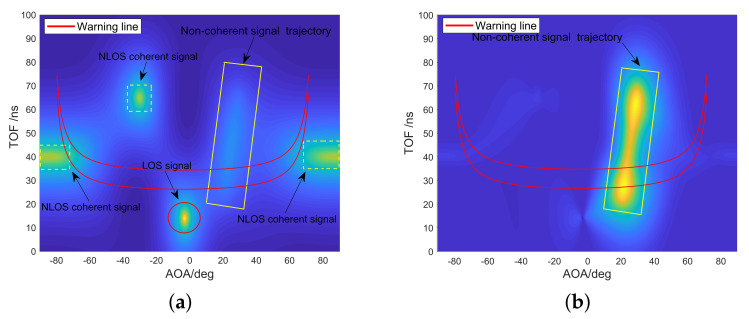
Pseudo spectrum of the foreign object detection and localization algorithm. (**a**) Without static clutter suppression. (**b**) With static clutter suppression.

After static clutter suppression, as shown in Figure 7, the movement trajectory of foreign object intrusion can be detected more clearly. To achieve available metro foreign object intrusion detection and alarm, it is also necessary to construct a warning area that affects the safety of the train in the scene and map the warning area to the pseudo spectrum. When a foreign object is detected entering or passing through the warning area, an alarm message is sent over the WiFi communication link. The width of the type B metro train is 2.8 m. In this paper, it is defined as a warning area where the track centerline extends 1.5 m to both sides.

(15) $$\left\{\begin{array}{c} \mathrm{AOA}=\arctan(y/x)\\ \mathrm{TOF}=2(\sqrt{x^{2}+y^{2}})/c \end{array}\right.$$

**Figure 8:**
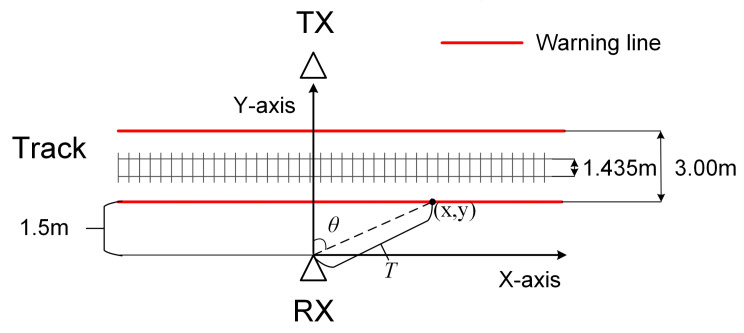
Warning line model.

The method of mapping the alarm area to the pseudo spectrum is shown in Figure 8 and Equation (15). In the rectangular coordinate system with the receiver as the origin, a point on the alarm line gets a coordinate with (x,y). In order to convert planar Cartesian coordinates (x,y) to pseudo spectrum coordinates (AOA,TOF), a warning line model is used to figure out the relationship between θ and (x,y), and the relationship between *T* and (x,y) in Figure 8 and Equation (15). After a geometric change, θ is the AOA between each point on the alarm line and the receiver, and *T* is the TOF converted by the distance between each point on the alarm line and the receiver.

Figure 7 is the visual output of a verification experiment of foreign object intrusion detection and static clutter suppression algorithms. Figure 7a shows the results of foreign object detection without static clutter suppression. It shows one direct path signal and three non-target reflection path signals, and the intrusion path signals of the foreign object are almost covered by these signals. After static clutter suppression, the static path is eliminated, and the trajectory of foreign object movement can be easily found. It can also be observed from Figure 7b that the trajectory of the foreign object intrusion crossed the warning area and eventually stayed near the warning area. The complete foreign object detection and localization algorithm is summarized in Algorithm 2.
**Algorithm 2** Foreign object detection and localization algorithm.**Input:**${\mathrm{CSI}}_{re}$ matrix after phase calibration output by Algorithm 1**Output:** 1 Foreign object Intrusion Alarm    2 Coordinates of the foreign object1:Demodulate coherence of the ${\mathrm{CSI}}_{re}$2:Run MUSIC algorithm,output AOA $\theta_{t}$3:Calculate $d_{j,t}$ based on the CSI energy attenuation model (Equation (13))4:Input coordinate$\left(x,y\right)=\left(d_{j,t}\cos\theta_{t},d_{j,t}\sin\theta_{t}\right)$5:**if** Foreign objects in the warning area **then**6: Intrusion Alarm7:**end if**8:Static Clutter suppression using Equation (14)9:**if** Dynamic trajectory pass throuh the warning area **then**10: Intrusion Alarm11:**end if**

### 4.4. Train Recognition Based on RCS

When the train passes by, it is necessary to distinguish between the train and the foreign object without causing a false alarm. Compared with foreign objects, trains have fixed and obvious physical features. Thus, we need a parameter representing such features to classify the trains and foreign objects.

The RCS is a physical quantity that measures the intensity of the echo generated by the target under the irradiation of electromagnetic waves. The definition of RCS is as follows:

(16) $$\sigma =4\pi \frac{P_{r}}{\frac{A_{r}}{d_{r}^{2}}}\frac{1}{\frac{P_{t}G_{t}}{4\pi d_{t}^{2}}}$$

where the $P_{r}$ is receiver power input and $P_{t}$ is the transmitter’s power output. $G_{r}$ and $G_{t}$ are the gain of the receiving antenna and the transmitting antenna, respectively. $d_{t}$ and $d_{r}$ are, respectively, the distances from the transmitting antenna to the target and the receiving antenna to the target. $A_{r}$ denotes the effective area of aperture of the receiving antenna:

(17) $$A_{r}=\frac{G_{r}\lambda^{2}}{4\pi }$$

where λ is the wavelength.

According to Equation (16), if the sensor parameters are given, and the distance between the sensor and the target is known, the RCS value of the target can be calculated based on the receiver power input $P_{r}$. Simultaneously, when electromagnetic waves radiate the target with the same frequency from different directions, the RCS is different. Besides, the shape and surface of the material will also significantly affect the RCS value of the target. Therefore, RCS is an essential property of the object. Therefore, if the RCS data set of a specific target (such as a train) can be constructed, the method of machine learning can be used to classify and identify the target. Based on this idea, we use MatLab to build a train RCS data set and use support vector machines (SVM) to classify trains and foreign objects. The results of the simulation are in Section 5.3.

## 5. Performance Evaluation

**Figure 9:**
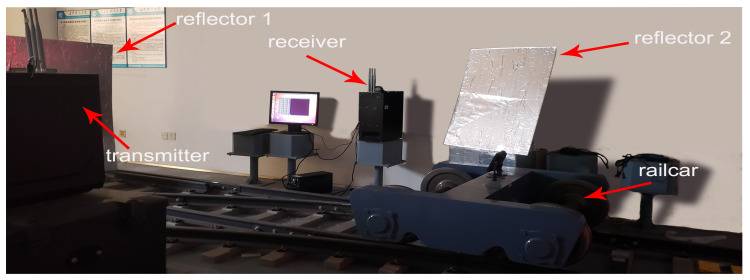
Photographs of the experimental scenario.

The Intel 5300 WiFi NIC is installed on the Dell OptiPlex 3050MT PC as the transmitter and receiver. The operating system of the PC is Ubuntu 10.04 LTS, and CSI Tool [10,11] is installed to obtain CSI information. The transmitter is equipped with one antenna, and the receiver is equipped with three antennas. The experimental environment is shown in Figure 9, a transmitter and a receiver are deployed on one side of the track. After the transmitter and receiver are deployed, the position of the transmitter is kept unchanged, and the angle between the two devices is measured as the input parameter of the fast phase calibration algorithm. In the experiment, the intruding foreign bodies are an adult man, a box with a size of 25 × 30 × 20 cm, and a rail car that could move at a maximum speed of 10 km/h. In order to reduce the difference between the experimental environment and the real tunnel scene as much as possible, in the experiment, we set up two additional reflection planes and wrapped the reflection plane with tin tin-foil to reduce the attenuation of the reflected signal. The specific parameters of the experiment are shown in Table 2.

### 5.1. Visualization and Performance of Detecting Foreign Object

Two metrics, positive detection probability (PDP) and negative detection probability (NDP), are defined to evaluate the foreign object detection performance of the proposed method. The PDP is the probability that a foreign object is successfully detected in the presence of a foreign object. The NDP is the probability that no false alarm will occur if there is no foreign object.

(18) $$PDP=\frac{p_{d}}{p_{r}}\times 100\%$$

(19) $$NDP=\frac{n_{d}}{n_{r}}\times 100\%$$

When there is a foreign object in the detection area, in a PDP evaluation experiment, $p_{d}$ is the number of experiments in which the algorithm detects the foreign object and $p_{r}$ is the total number of experiments. When there is no foreign object in the detection area, it is an NDP evaluation experiment, wherein $n_{d}$ is the number of experiments in which the algorithm has not detected foreign objects and $n_{r}$ is the total number of experiments.

#### 5.1.1. Comparison of Information Theory Criterion

**Figure 10:**
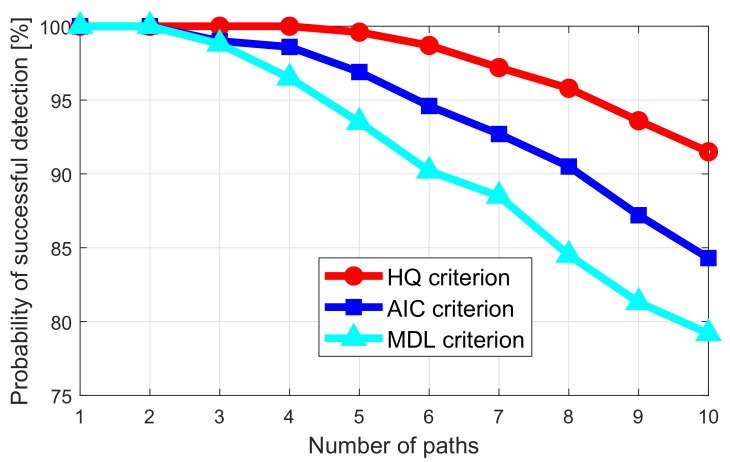
Comparison of the probability of successful detection regarding the number of paths with different information theory criteria.

The accurate estimation of the number of reflected signal paths in the tunnel is the basis for the detection of foreign objects in the subway. In the case of SNR of 1dB, compare the accuracy of path number detection of the three criteria of HQ, MDL, and AIC. The results are shown in Figure 10. When the number of paths is less than four, all three criteria can achieve a detection accuracy rate of more than 95%. When the number of paths is greater than seven, this is normal in real indoor and tunnel environments. The detection accuracy of the path number of the HQ criterion is significantly better than the other two. The higher the number of paths, the greater the advantage of the HQ criterion. Therefore, in the following experiments, we use the HQ criterion to realize the path number estimation.

#### 5.1.2. Visualization of Detecting a Foreign Object

**Figure 11:**
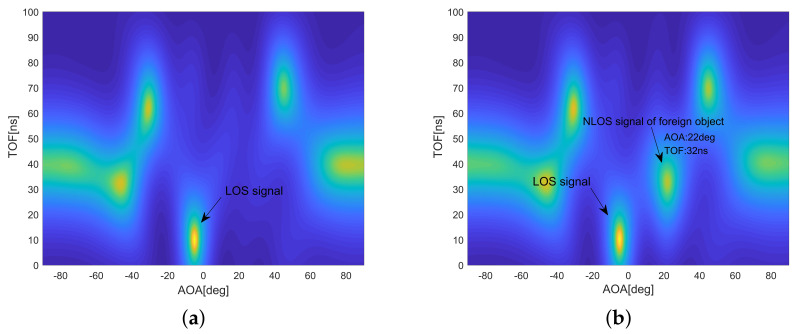
Visualization results of foreign object detection. (**a**) Without foreign object. (**b**) With foreign object.

We designed a heat map visualization tool based on the MUSIC pseudo-spectrum to visually observe the invasion process of foreign objects and calculate the location information of foreign objects. In the experimental scenario, in addition to the LOS signal between the transceivers, objects in the detection area will reflect the signal sent by the transmitter. These signals form peaks (highlighted areas) in the heat map. Figure 11a is the experimental result before foreign body intrusion. In addition to the LOS signal, there are five reflected signals. As shown in Figure 11b, after the foreign object invades, the reflected signal increases by one, and obviously, the increased reflected signal is caused by the foreign object. By calculating the coordinates of the signal in the thermal map, the positioning of the foreign object can be achieved.

#### 5.1.3. Impact of Spatial Smoothing and Phase Calibration on AOA Estimation

**Figure 12:**
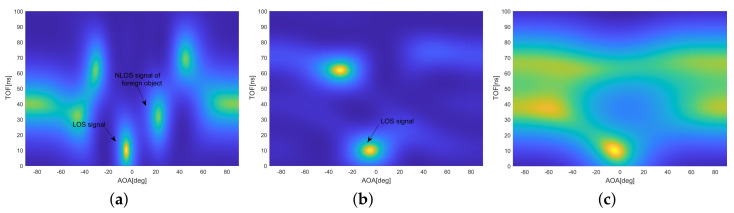
Impact of spatial smoothing and phase calibration on AOA estimation. (**a**) With spatial smoothing and phase calibration. (**b**) Without spatial smoothing. (**c**) Without phase calibration.

In order to verify the effect of spatial smoothing on AOA estimation, a comparative experiment was carried out in the presence or absence of coherent signals. As shown in Figure 12a, there were seven coherent signals, including one LOS signal and one NLOS signal of a foreign object on MUSIC pseudo-spectrum. It is clear to estimate the AOA of each coherent signal with spatial smoothing, and only the LOS coherent signal was estimated without spatial smoothing in Figure 12b. Therefore, in the case of coherent signals, spatial smoothing must be used to decoherent to ensure the accuracy of AOA estimation.

Compared to our conference paper [26], fast phase calibration is one of the core improvements in this paper. In order to test the effectiveness and importance of the phase calibration algorithm, Figure 12c shows the experimental results without pre-phase calibration. Compared with Figure 12a, which has the correct result, Figure 12c has severe distortion in the calculation of the signal number and the foreign object positioning information.

#### 5.1.4. Impact of Distance to Receiver on Detection Probability

**Figure 13:**
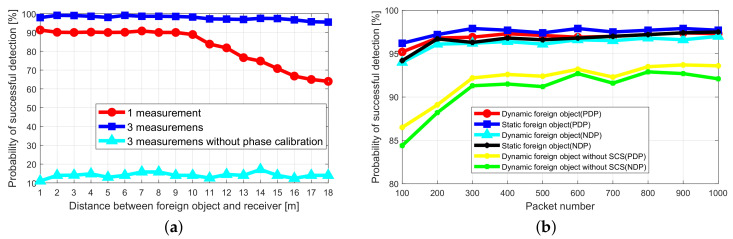
Experimental results of foreign object detection probability. (**a**) Impact of Distance to Receiver and the number of measurements on Detection Probability. (**b**) Impact of Packet Number on Detection Probability.

In order to verify the coverage of the foreign object detection, the foreign object is placed at a different distance from the receiver, and the verification is performed by analyzing the success probability of the foreign object detection. For a single measurement, the transmitter sends ten packets to the receiver, and the packet sending interval is 10 ms. As shown in Figure 13a, in the case of one measurement, when the distance between the foreign object and the receiver is less than 10 m, the PDP is greater than 90%. When the distance is greater than 10 m, the PDP gradually decreases. When the distance is 18 m, the PDP is 63.9%. When the position of the foreign object is not changed and three consecutive measurements are adopted, the PDP of more than 95.8% can be achieved within 18 m. The experiment shows that the system can meet the coverage of 18 m, and under the premise of ensuring a higher PDP, it can complete the detection of foreign objects in 0.4 s.

While keeping the experimental settings unchanged, we performed three consecutive measurements without phase calibration. In Figure 13a, the light blue curve is the experimental result, and the PDP is always below 20%. This is due to the abnormal phase offset of the RF chains. Therefore, whether the fast phase calibration algorithm is used to preprocess the CSI data has a great influence on the probability of successful detection of foreign objects.

#### 5.1.5. Impact of Packet Number on Detection Probability

In order to verify the impact of the number of packets on the performance of foreign object detection, different numbers of data packets are sent to evaluate the PDP and NDP of foreign object detection. As shown in Figure 13b, no matter whether the foreign object is stationary or moving, when the number of packets exceeds 200, it can achieve a PDP of 96.8% and a NDP of 96.1%. The experiment shows that it is easy to achieve the higher PDP and NDP by appropriately increasing the interval of sending packets and increasing the number of packets. For example, the packet sending interval is 1 ms, and the number of sending packets is 300, which can guarantee high success rate detection within 0.4 s, and can satisfy the requirements of the application.

To test the effectiveness of the static clutter suppression (SCS) algorithm, in Figure 13b, the yellow and green curves are the experimental results without the SCS algorithm. Both PDP and NDP are reduced. Especially when the number of data packets is relatively small, the SCS algorithm’s impact is more significant. When the number of data packets is 100, PDP and NDP drop to 86.5% and 84.1%, respectively. This shows that the SCS algorithm can increase the probability of successful detection of dynamic foreign objects. Especially when the number of data packets is relatively small, the role of the SCS algorithm is more prominent.

### 5.2. Performance of Localizing Foreign Object

In order to evaluate the localization performance of the proposed method, the proposed method was compared with the dynamic-MUSIC algorithm [34], the accuracy of the object angle estimation was tested, and the effects of the different object moving speeds, and different sending quantities on the localization accuracy were verified. Considering that the object cannot be regarded as a point, if the error of the estimated position is within 45 cm, it is considered that there is no localization error. The experimental results in this section are based on this premise.

**Figure 14:**
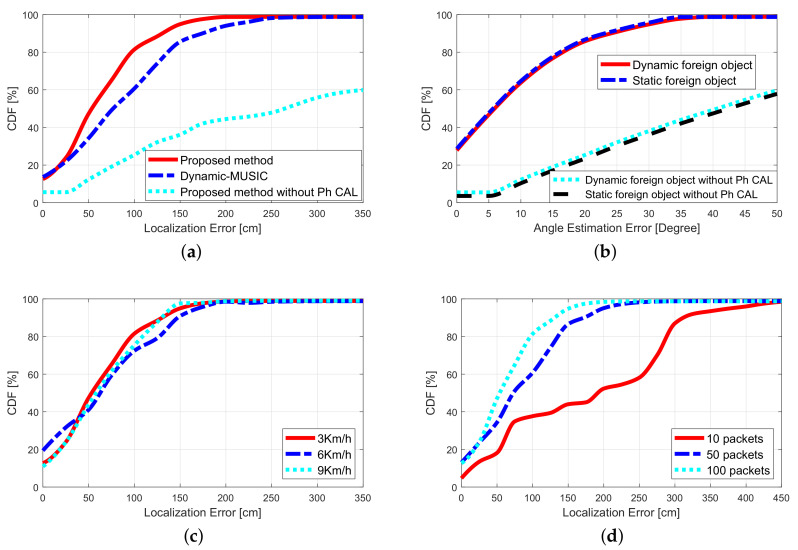
Experimental results of foreign object localization performance. (**a**) Comparison with Other Algorithms. (**b**) Performance of Angle Estimation. (**c**) Impact of Speed on Localization. (**d**) Impact of Packet Number on Localization.

At the same time, in order to highlight the improvement of this article compared to the conference paper we published, Figure 14a,b also added the experimental results without phase calibration as a comparison.

#### 5.2.1. Comparison with Other Algorithms

A position estimation is performed on a foreign object moving at a speed of 3 km/h. The comparative experimental results of the proposed method and the dynamic-MUSIC algorithm are shown in Figure 14a. The experimental results show that the median localization error of the proposed algorithm is 60.5 cm, and the dynamic-Music algorithm is 74.9 cm. The localization accuracy of the proposed algorithm is higher than the dynamic-MUSIC algorithm. The reason may be that the proposed fast phase calibration method has better phase calibration performance.

To test the impact of the fast phase calibration algorithm on the localization accuracy, a foreign object positioning experiment was conducted without performing phase calibration on the CSI data. In Figure 14a, the experimental results show that the median localization error is 296.2 cm and has intense volatility. The localization error is substantial, and it is almost unavailable in practical applications.

#### 5.2.2. Performance of Angle Estimation

Figure 14b shows the cumulative distribution function (CDF) of the angle estimation error of the foreign object. The median errors of the angle estimation of the foreign object in the stationary state and the moving state (speed 6 km/h) are 5.1 degrees and 6.6 degrees, respectively. Experiments show that the motion state of a foreign object has little effect on the accuracy of angle estimation.

To test the impact of the fast phase calibration algorithm on the accuracy of angle calculation, without calculating the phase of the CSI data, angle calculation experiments were performed on dynamic and static foreign objects. As shown in Figure 14b, the experimental results show that the average error of the angle estimation is 40 degrees, and it is almost impossible to achieve useful angle estimation.

#### 5.2.3. Impact of Speed on Localization

In order to verify the impact of the speed of the foreign object on localization accuracy. We set the rail car to pass the detection zone at speeds of 3, 6, and 9 km/h, respectively. The test results are shown in Figure 14c, and the median localization errors are 61.2, 64.6, and 67.1 cm respectively. It can be seen that when the moving speed of the foreign object is below 9 km/h, the speed has little effect on the localization accuracy.

#### 5.2.4. Impact of Packet Number on Localization

It can be inferred from Equation (10) that the number of packets has an effect on the accuracy of foreign object localization. In order to test this effect, keep the foreign body in a static state, and test the foreign object localization accuracy when transmitting 10 packets, 50 packets, and 100 packets, respectively. The test results are shown in Figure 14d. The median localization errors are 193.1 cm, 76.9 cm and 60.1 cm, respectively. The experimental results show that the number of packets has a certain effect on the localization accuracy of foreign objects, but the number of packets exceeds 100, and the impact of further increasing the number of packets on the localization accuracy is not obvious. Therefore, if the number of packages is more than 100, satisfactory localization performance of foreign objects can be obtained.

### 5.3. Simulation of Train Recognition

In order to verify the feasibility of the RCS-based train and foreign object classification method, we used MatLab to build a simulation RCS dataset of trains and foreign objects. The train size is set to 20 × 2.8 × 3.8 m, and the running speed is 100 km/h. The foreign objects are set as regular tetrahedrons with volumes 0.5, 14.6, and 18 m${}^{3}$, respectively.

**Figure 15:**
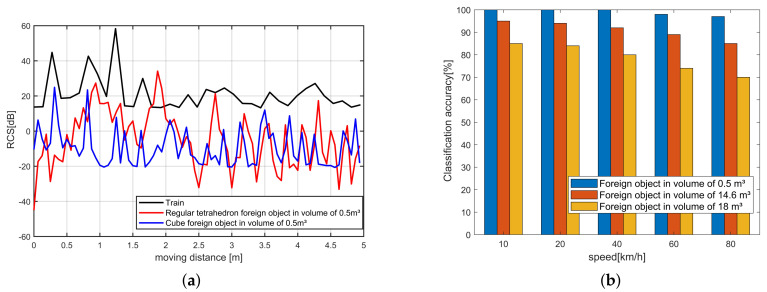
Simulation results of classification accuracy. (**a**) RCS of train and foreign objects. (**b**) Impact of train’s speed and foreign object’s volume on classification accuracy.

Figure 15a is the RCS change curve of the train and the foreign object with a volume of 0.5 m driving through the receiver at a speed of 100 km/h, respectively. It can be seen from the figure that the RCS change curve of the train and the foreign body has a significant difference, this is because the reflection cross-section of the train is much larger than the foreign body. Collect the RCS change curve to construct the RCS data set, and train the SVM classifier to classify and identify trains and foreign objects. The classification accuracy rate is shown in Figure 15b. For normal-sized foreign objects, the classification accuracy rate exceeds 95%. Simulation results prove that the classification of trains and foreign objects based on RCS is feasible.

## 6. Conclusions

In this paper, we have achieved an accurate intrusion detection of foreign objects in the metro tunnel environment using commercial WiFi NICs based on the fast phase calibration algorithm, which solves the CSI phase abnormality Induced by the clock non-synchronization between the radio oscillators under each antenna of the WiFi NIC. The experimental results show that the proposed fast phase calibration algorithm can rapidly and accurately correct phase anomalies, and the MUSIC algorithm combined with static clutter suppression can achieve higher foreign object detection probability and foreign object localization accuracy.

The accuracy of distance estimation from foreign objects is an important factor affecting the performance of the proposed method. One of the future work is to solve the problem of clock synchronization between the receiver and transmitter to improve the accuracy of distance estimation of foreign objects. Another potential improvement of the proposed algorithm is to consider the use of machine learning algorithms to analyze foreign object features (such as radar cross section (RCS)) to achieve the material or size recognition of foreign objects.

## Figures and Tables

**Table 1:** The mapping relationship between the logical RF chains and the antenna ports.

${\mathrm{Antenna}}_{1}$	${\mathrm{Antenna}}_{2}$ **(Mid Antenna)**	${\mathrm{Antenna}}_{3}$
$RF\;Chain_{1}$	$RF\;Chain_{3}$	$RF\;Chain_{2}$

**Table 2:** Optimal results for two different load cases.

Experimental Facilities	Parameters	Value
Rail	width (m)	1.435
	height (m)	0.176
Transceiver	NIC	Intel 5300
	distance (m)	4.5
	angle (${}^{\circ }$)	0
	frequency (GHz)	5
	distance between antennas (cm)	2.6
	number of OFDM carrier	30

**Figure A1:**
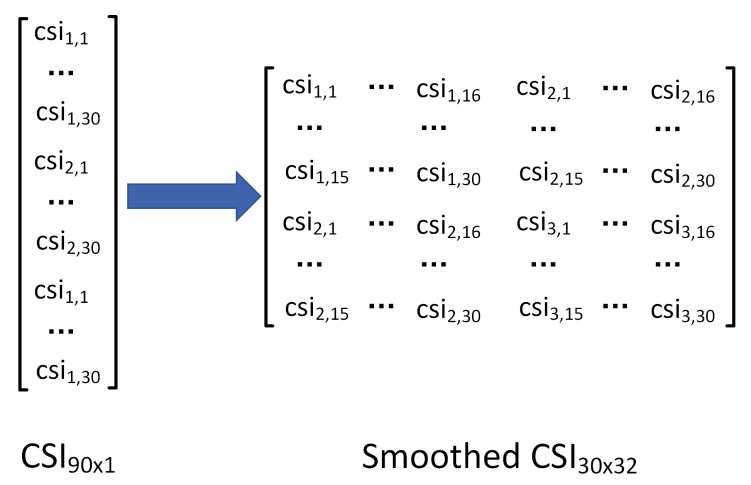
Construction of smoothed CSI matrix.

## Abbreviations

The Symbol Notation Used in Algorithms and Models:

**Symbol**	**Description**
$\tau_{S}$	the time delay of transceiver caused by clock unsynchronization
$\phi_{s}$	the phase offset caused by clock unsynchronization
$f_{\delta }$	the frequency spacing between two consecutive subcarriers
*n*	the subcarrier index
*d*	the antenna distance of transceiver
θ	the signal incident angle to reciever’s antennas
*f*	the center frequency of subcarrier
*c*	the speed of light
*m*	the antenna index
*i*	the packet index
$\phi_{i}\left(m,n\right)$	the phase of the *n*th subcarrier of the *m*th antenna in the *i*th packet
$\tau_{s,i}$	the time delay of the *i*th packet
ρ	the linear fit variable
β	the linear fit variable
M	the number of antennas in transmitter or receiver
N	the number of subcarriers
${\hat{\tau }}_{s,i}$	the time delay of best linear fit
${\hat{\phi }}_{i}\left(m,n\right)$	the sanitized phase of the *n*th subcarrier of the *m*th antenna in the *i*th packet
*L*	the number of signal propagation paths
*k*	the index of signal propagation paths
$S_{L}\left(t\right)$	the transmitting signal of *L*th propagation paths
A(θ)	the ideal steering matrix for transmitting signals
λ	the wavelength of transmitting signals
$\phi_{k}$	the phase offset of the transmitted signal on the first antenna for *k*th path
$S_{k}\left(t\right)$	the transmitting signal of *k*th propagation paths
$s_{N}\left(t\right)$	the transmitting signal of *n*th subcarrier
$\psi_{k}$	the phase difference between subcarriers in *k*th propagation paths
$\tau_{k}$	the TOF between subcarriers in *k*th propagation paths
$\Phi_{\theta k}$	$e^{-j\phi_{k}}$
$\psi_{\tau k}$	$e^{-j\psi_{k}}$
n(t)	the signal noise
$A\left(\theta_{k},\tau_{k}\right)$	the steering matrix of the *k*th path
$e^{{-j\delta }_{m}}$	the phase offset caused by the clock unsynchronization of intel 5300 WiFi NIC
O(δ)	the phase offset matrix on the antenna array
${\mathrm{CSI}}_{re}$	the real CSI matrix
$\phi_{perm3,n}$	the phase of mid antenna on $n^{\mathrm{th}}$ subcarrier
ζ	the performance evaluation parameter of the fast phase calibration
$n_{t}$	the data size
$T(n_{t})$	the execution time of the code
$f(n_{t})$	the total execution times
X	the CSI matrix
R	the covariance matrix of CSI phase
$U_{S}$	the signal subspace
$U_{N}$	the noise subspace
J(l)	the generalized expression of the information theory criterion
L(l)	the log-likelihood function
P(l)	the penalty function
*l*	the number of signal sources to be estimated
*S*	the number of samples
Λ(l)	the likelihood function
$P_{MUSIC,t}$	the spectrum for AOA and TOF
${\bar{P}}_{MUSIC,t}$	the mean value of the spectrum for AOA and TOF
${\hat{P}}_{MUSIC,t}$	the suppressed spectrum for AOA and TOF
*T*	the times number of spectrum estimation
$R_{ij}$	the amplitude attenuation of CSI from transmitter *i* to receiver *j*
$L_{ij}$	the propagation attenuation of CSI from transmitter *i* to receiver *j*
$D_{ij}$	the diffraction attenuation of CSI from transmitter *i* to receiver *j*
$A_{t}$	the target absorption attenuation
$\theta_{t}$	the angle of the foreign object to the receiver
$d_{i,t}$	the distance from transmitter to the foreign object
$d_{j,t}$	the distance from the foreign object to the receiver
*x*	x coordinate of the foreign object
*y*	y coordinate of the foreign object
$p_{d}$	the number of times that there is a foreign object detected in all the detection times
$p_{r}$	the number of times a foreign object actually exists in all the detection times
$n_{d}$	the number of times that there is no foreign objects detected in all the detection times
$n_{r}$	the number of times a foreign object actually doesn’t exist in all the detection times
$P_{r}$	the receiver power input
$P_{t}$	the transmitter power output
$G_{r}$	the gain of the receiving antenna
$G_{t}$	the gain of the transmitting antenna
$d_{t}$	the distance from the transmitting antenna to the target
$d_{r}$	the distance from the receiving antenna to the target
$A_{r}$	the effective area of aperture of the receiving antenna

## Appendix A. Phase Sanitization Algorithm

To eliminate the additional phase induced by clock non-synchronization between a WiFi transmitter-receiver pair, we use the method of linear fitting in literature [18] to correct it. Let the phase of the *n*th subcarrier of the *m*th antenna in the *i*th packet be $\phi_{i}\left(m,n\right)$; $\tau_{s,i}$ is the time delay of the *i*th packet. We remove the CSI phase best linear fit term of the packet *i* by using a phase sanitization algorithm to obtain the sanitized phase. The CSI phase best linear fit is:

(A1) $${\hat{\tau }}_{s,i}=\underset{\rho ,\beta }{\arg\min}\sum_{m,n=1}^{M,N}{\left(\phi_{i}\left(m,n\right)+2\pi f_{\delta }\left(n-1\right)\rho +\beta \right)}^{2}$$

where M and N are the total number of array antennas and the number of subcarriers, respectively; both ρ and β are linear fit variables. Then, the sanitized CSI phase is:

(A2) $${\hat{\phi }}_{i}\left(m,n\right)=\phi_{i}\left(m,n\right)+2\pi f_{\delta }\left(n-1\right){\hat{\tau }}_{s,i}$$

## Appendix B. Information Theory Criterion

The information theory criterion J(l) consists of the log-likelihood function and the penalty function:

(A3) J(l)=L(l)+P(l)

where L(l) is the log-likelihood function and P(l) is the penalty function. By choosing different L(l) and P(l), we can get different criteria. For example, the EDC criterion can be expressed as:

(A4) EDC(l)=S(M−l)lnΛ(l)+l(2M−l)C(S)

*l* is the number of signal sources to be estimated; *M* is the number of sensors (in this case antennas). *S* is the number of samples in Equation (A4), where Λ(l) is the likelihood function:

(A5) $$\Lambda \left(l\right)=\frac{\frac{1}{M-l}\sum_{i=l+1}^{M}\lambda_{i}}{{\left(\prod_{i=l+1}^{M}\lambda_{i}\right)}^{\frac{1}{M-l}}}$$

And the C(S) in Equation (A4) must meet two conditions to have the estimation consistent:

(A6) $$\lim_{S\to \infty }\left(C\left(S\right)/S\right)=0$$

(A7) $$\lim_{S\to \infty }\left(C\left(S\right)/\ln\ln S\right)=\infty$$

We can get AIC, MDL, and HQ criterion when the C(S) in Equation (A4) is selected as 1, (lnS)/2 and (lnlnS)/2:

(A8) AIC(l)=S(M−l)lnΛ(l)+l(2M−l)

(A9) MDL(l)=S(M−l)lnΛ(l)+l(2M−l)(lnS)/2

(A10) HQ(l)=S(M−l)lnΛ(l)+l(2M−l)(lnlnS)/2

## Appendix C. CSI Spatial Smoothing Matrix

As shown in Figure A1, we construct the raw CSI matrix (obtained from CSI Tool) as a smoothed CSI matrix. After spatial smoothing, the number of sensors is reduced from 90 to 30. Obviously, the utilization efficiency of the sensor has decreased, and in theory, the upper limit of the number of reflection paths that can be detected is also reduced.
